# Steeper memory decline after COVID-19 lockdown measures

**DOI:** 10.1186/s13195-023-01226-5

**Published:** 2023-04-15

**Authors:** Els D. Bakker, Stéphanie L. van der Pas, Marissa D. Zwan, Freek Gillissen, Femke H. Bouwman, Philip Scheltens, Wiesje M. van der Flier, Ingrid S. van Maurik

**Affiliations:** 1 Alzheimer Center Amsterdam, Neurology, Vrije Universiteit Amsterdam, Amsterdam UMC Location VUmc, De Boelelaan 1118, Amsterdam, The Netherlands; 2 Amsterdam Neuroscience, Neurodegeneration, Amsterdam, The Netherlands; 3 Amsterdam UMC Location Vrije Universiteit Amsterdam, Epidemiology and Data Science, De Boelelaan 1117, Amsterdam, The Netherlands; 4 Amsterdam Public Health, Methodology, Amsterdam, The Netherlands

**Keywords:** COVID-19, Lockdown, Cognitive decline, Dementia, MCI, Subjective cognitive decline

## Abstract

**Background:**

During COVID-19 lockdown measures, memory clinic patients reported worries for faster cognitive decline, due to loss of structure and feelings of loneliness and depression. We aimed to investigate the impact of the COVID-19 lockdown on rate of cognitive decline in a mixed memory clinic population, compared to matched historical controls.

**Methods:**

We included patients who visited Alzheimer Center Amsterdam 6 months to 1 week before the first Dutch COVID-19 lockdown, and had a second visit 1 year later, after this lockdown period (*n* = 113; 66 ± 7 years old; 30% female; *n* = 55 dementia, *n* = 31 mild cognitive impairment (MCI), *n* = 18 subjective cognitive decline (SCD), *n* = 9 postponed diagnosis). Historical controls (visit in 2016/2017 and second visit 1 year later (*n* = 640)) were matched 1:1 to lockdown patients by optimal Mahalanobis distance matching (both groups *n* = 113). Groups were well matched. Differences between lockdown patients and historical controls over time in Mini-Mental State Examination, Trail Making Test part A and B, Rey-Auditory Verbal Learning Test (RAVLT) immediate and delayed recall, and category fluency scores were analyzed using linear mixed effect models with random intercepts. We examined differences in rate of cognitive decline between whole groups, and after stratification in SCD, MCI, and dementia separately.

**Results:**

Lockdown patients had a faster rate of memory decline compared to controls on both RAVLT immediate [*B*(SE) =  − 2.62 (1.07), *p* = 0.015] and delayed recall [*B*(SE) =  − 1.07 (0.34), *p* = 0.002]. Stratification by syndrome diagnosis showed that this effect was largely attributable to non-demented participants, as we observed faster memory decline during lockdown in SCD and MCI (RAVLT immediate [SCD: *B*(SE) =  − 6.85 (2.97), *p* = 0.027; MCI: *B*(SE) =  − 6.14 (1.78), *p* = 0.001] and delayed recall [SCD: *B*(SE) =  − 2.45 (1.11), *p* = 0.035; MCI: *B*(SE) =  − 1.50 (0.51), *p* = 0.005]), but not in dementia.

**Conclusion:**

Memory clinic patients, specifically in pre-dementia stages, showed faster memory decline during COVID-19 lockdown, providing evidence that lockdown regulations had a deleterious effect on brain health. In individuals that may have been able to deal with accumulating, subclinical neuropathology under normal and structured circumstances, the additional stress of lockdown regulations may have acted as a “second hit,” resulting in less beneficial disease trajectory.

**Supplementary Information:**

The online version contains supplementary material available at 10.1186/s13195-023-01226-5.

## Background

For people with cognitive impairment or dementia, lockdown restrictions led to disruption in formal and informal support systems, having a major influence on patients’ daily lives [1–3]. In earlier survey studies, we showed that during Dutch lockdown, the restrictions on social contact made it difficult for informal support networks to help their loved ones [3, 4]. In addition, non-acute health care appointments and home care were down-graded, day care facilities were closed, and contact with case managers and volunteers was decreased [3].

This loss of structure and support in daily life could imply a risk of faster cognitive decline in patients with cognitive impairment, especially in pre-dementia stages. In a substantial proportion of individuals with mild cognitive impairment (MCI), and to a smaller extent subjective cognitive decline (SCD), the underlying cause of cognitive complaints is neurodegenerative disease [5, 6]. While individuals may be resilient to this accumulating pathology in structured situations, the loss of structure in combination with high levels of stress imposed by the COVID-19 pandemic and associated lockdown measures could tip patients over the edge, causing the underlying disease to manifest sooner. Previous survey studies show that both patients and caregivers reported worries for steeper cognitive decline during the COVID-19 pandemic [3, 4, 7, 8]. These worries were not limited to patients with dementia, but were also reported by substantial proportions of patients with MCI and people with SCD [3, 4, 9].

Two small previous studies reported a larger decline in Mini-Mental State Examination (MMSE) during lockdown than before in MCI and dementia [10, 11]. However, there were some methodological concerns, particularly as a matched control group was lacking, and the focus was mainly on the dementia stage while memory clinic populations are much broader than that. Therefore, we aimed to investigate the impact of the COVID-19 lockdown on cognitive decline over time in a mixed memory clinic population, including SCD, MCI, and dementia patients, and compared them to matched historical controls.

## Methods

### Participants

In this matched longitudinal study, we included patients from the Amsterdam Dementia Cohort (ADC) [12, 13]. We selected two groups of patients: (1) Lockdown patients: patients with a visit at the memory clinic 6 months to 1 week before the first COVID-19 lockdown in the Netherland (mid-March 2020), and a second visit approximately 1 year later, after this lockdown period. Inclusion criteria was complete data on cognitive tests. The lockdown group included patients with a diagnosis of SCD (*n* = 18), MCI (*n* = 31), dementia (*n* = 26 Alzheimer’s dementia (AD), *n* = 23 dementia with Lewy Bodies (DLB), *n* = 6 frontotemporal dementia (FTD) or primary progressive aphasia (PPA)), or a postponed diagnosis (*n* = 9). We excluded one patient with a psychiatric diagnosis, because of limited power for this diagnosis group. In total, *n* = 113 lockdown patients were included. (2) Historical control patients: patients with a visit at the memory clinic in 2016 or 2017, and a second visit 1 year later. We excluded *n* = 51 patients with another diagnosis than present in the lockdown patient group (vascular dementia diagnosis, psychiatric diagnosis, or other neurological diagnosis). In total, *n* = 640 eligible historical control patients were identified (*n* = 236 SCD, *n* = 139 MCI, *n* = 245 dementia, *n* = 20 postponed diagnosis).

All patients underwent cognitive screening at Alzheimer Center Amsterdam, which is a tertiary memory clinic. In general, patients referred to Alzheimer Center Amsterdam undergo a standardized dementia baseline diagnostic work-up [12, 13]. This baseline diagnostic work-up consisted of neurological, physical, and neuropsychological evaluation; magnetic resonance imaging (MRI); laboratory tests; and lumbar puncture for cerebrospinal fluid (CSF) measurement. After the baseline diagnostic work-up, clinical diagnosis was made in a multi-disciplinary meeting. Patients were diagnosed according to the National Institute on Aging-Alzheimer’s Association (NIA-AA) criteria for MCI and AD dementia, the current consensus criteria for DLB, or the diagnostic criteria for FTD [5, 14–18]. A diagnosis SCD was made when the patient presented with cognitive complaints, but had normal clinical and cognitive test results and did not meet the criteria for MCI, dementia, or other neurological or psychiatric conditions [19]. When a clinical diagnosis remained unclear after the baseline diagnostic work-up and multidisciplinary meeting, the diagnosis was labeled as postponed diagnosis. Patients were invited for annual follow-up visits which include a neurological, physical, and neuropsychological evaluation. For the current study, we selected all patients who had a visit, either a baseline diagnostic work-up or follow-up visit, during the eligible time windows (described above). Of note, patients could therefore have a baseline visit or a follow-up visit selected as first measurement in the current study. All patients lived at home at the time of their visit to the memory clinic.

If patients reported a COVID-19 infection to the assessing physician, this information was included in the physician’s medical letter in the electronic patient file.

### Cognitive outcomes

We used the following cognitive tests as outcome measures: Mini-Mental State Examination (MMSE; global cognition), Trail Making Test (TMT) part A (attention and speed) and part B (executive functioning), Rey-Auditory Verbal Learning Test (RAVLT) immediate and delayed recall (memory), and category fluency (language) [20–22]. These cognitive tests are included in the yearly neuropsychological follow-up assessment of the Alzheimer Center Amsterdam [12, 13].

### Matching procedure

**Fig. 1:**
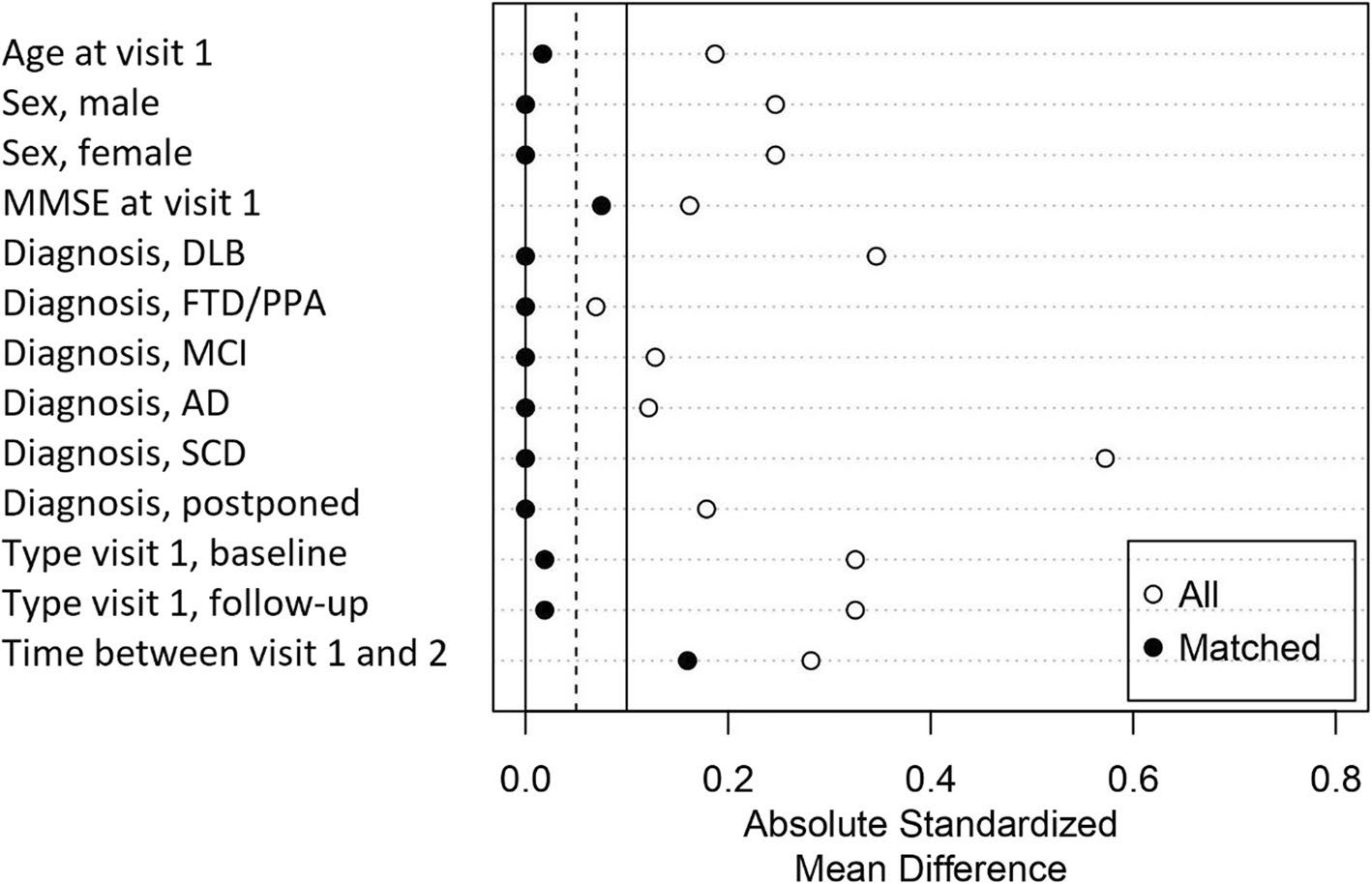
Absolute standardized mean differences for all matching variables, before (white dots) and after (black dots) optimal Mahalanobis distance matching. AD, Alzheimer’s dementia; DLB, dementia with Lewy bodies; FTD, frontotemporal dementia; MCI, mild cognitive impairment; MMSE, Mini-Mental State Examination; PPA, primary progressive aphasia; SCD, subjective cognitive decline

The matching analyses were carried out in R Studio 4.2.0, with package MatchIt [23]. Historical control patients were matched 1:1 to lockdown patients by several matching methods based on Mahalanobis distance: nearest neighbor matching, optimal matching, full matching, and genetic matching [24, 25]. Of these algorithms, 1:1 optimal Mahalanobis distance matching gave the best overall balance. Variables used for matching were age, sex, type of first visit (baseline or follow-up), diagnosis at first visit, MMSE score at first visit, and time between first and second visit. Prior to matching, all variables were inspected on completeness and similarity of distribution between the lockdown and historical control group (Table e-1, e-2 and e-3, Figure e-1, e-2 and e-3 in the supplement). Before and after matching, balance was assessed based on absolute standardized mean difference. Matching led to balanced groups with absolute standardized mean differences under 0.1 for almost all matching variables; see Fig. 1. Slight imbalance was present for the matching variable time between visits (absolute standardized mean difference of 0.16). Further inspection showed that the time between visits was on average 1 month longer in lockdown patients (14 months), compared to matched historical controls (13 months). This difference was not deemed relevant, and the matched set was considered adequately balanced on the matching covariates.

### Statistical analyses

We used descriptive statistics to report means and frequencies. We used linear mixed effect models (LMM) to investigate the effect of lockdown slopes of MMSE, TMT part A and B, RAVLT immediate and delayed recall, and category fluency. In the model, terms for time (0 = visit 1, 1 = visit 2), group (0 = historical control patients, 1 = lockdown patients), and interaction between time and group were added. LMM analyses were performed on matched groups. In an additional set of analyses, we repeated the LMM analyses on the entire cohort (without matching). Subsequently, we performed the LMM analyses stratified by syndrome diagnosis: SCD, MCI, and dementia. Due to the small number of patients with a postponed diagnosis, we did not include these patients in the stratified LMM analyses. Lastly, we performed a sensitivity analysis, in which we performed the LMM analyses for amyloid positive patients only. All analyses were carried out in SPSS Statistics version 28. *P* value of < 0.05 was considered significant.

## Results

### Descriptive statistics

Demographic and clinical characteristics of matched lockdown patients and historical control patients are summarized in Table 1. The lockdown patients and historical controls were similar on age, sex, MMSE, years of education, diagnosis, type of first visit, and time between first and second visit. Amyloid status was available for most patients (lockdown patients: *n* = 79 (70%; A + : *n* = 51 (65%)); historical controls: *n* = 90 (80%; A + : *n* = 48 (53%))). Characteristics of the whole historical control group can be found in the supplemental material (Table e-1).

**Table 1:** Demographic characteristics of lockdown patients and matched historical control patients

	**Lockdown patients**	**Matched historical controls**
	*n* = 113 (100%)	*n* = 113 (100%)
Age in years	66 ± 7	66 ± 7
Sex, female	*n* = 34 (30%)	*n* = 34 (30%)
MMSE at visit 1	25 ± 3	25 ± 3
Years of education	12 ± 3	12 ± 3
Diagnosis at visit 1		
SCD	*n* = 18 (16%)	*n* = 18 (16%)
MCI	*n* = 31 (27%)	*n* = 31 (27%)
AD	*n* = 26 (23%)	*n* = 26 (23%)
FTD or PPA	*n* = 6 (5%)	*n* = 6 (5%)
DLB	*n* = 23 (20%)	*n* = 23 (20%)
Postponed diagnosis	*n* = 9 (8%)	*n* = 9 (8%)
Visit 1 type		
Baseline	*n* = 36 (32%)	*n* = 37 (33%)
Follow-up	*n* = 77 (68%)	*n* = 76 (67%)
Time between visit 1 and visit 2 (in years)	1.2 ± 0.3	1.1 ± 0.3

*AD* Alzheimer’s dementia, *DLB* Dementia with Lewy bodies, *FTD* Frontotemporal dementia, *MCI* Mild cognitive impairment, *MMSE* Mini-Mental State Examination, *PPA* Primary progressive aphasia, *SCD* Subjective cognitive decline

Only two (2%) lockdown patients reported to their assessing physician that they had been infected with COVID-19; none of them reported a hospitalization due to COVID-19 infection.

### Cognitive decline in lockdown patients and historical controls

**Fig. 2:**
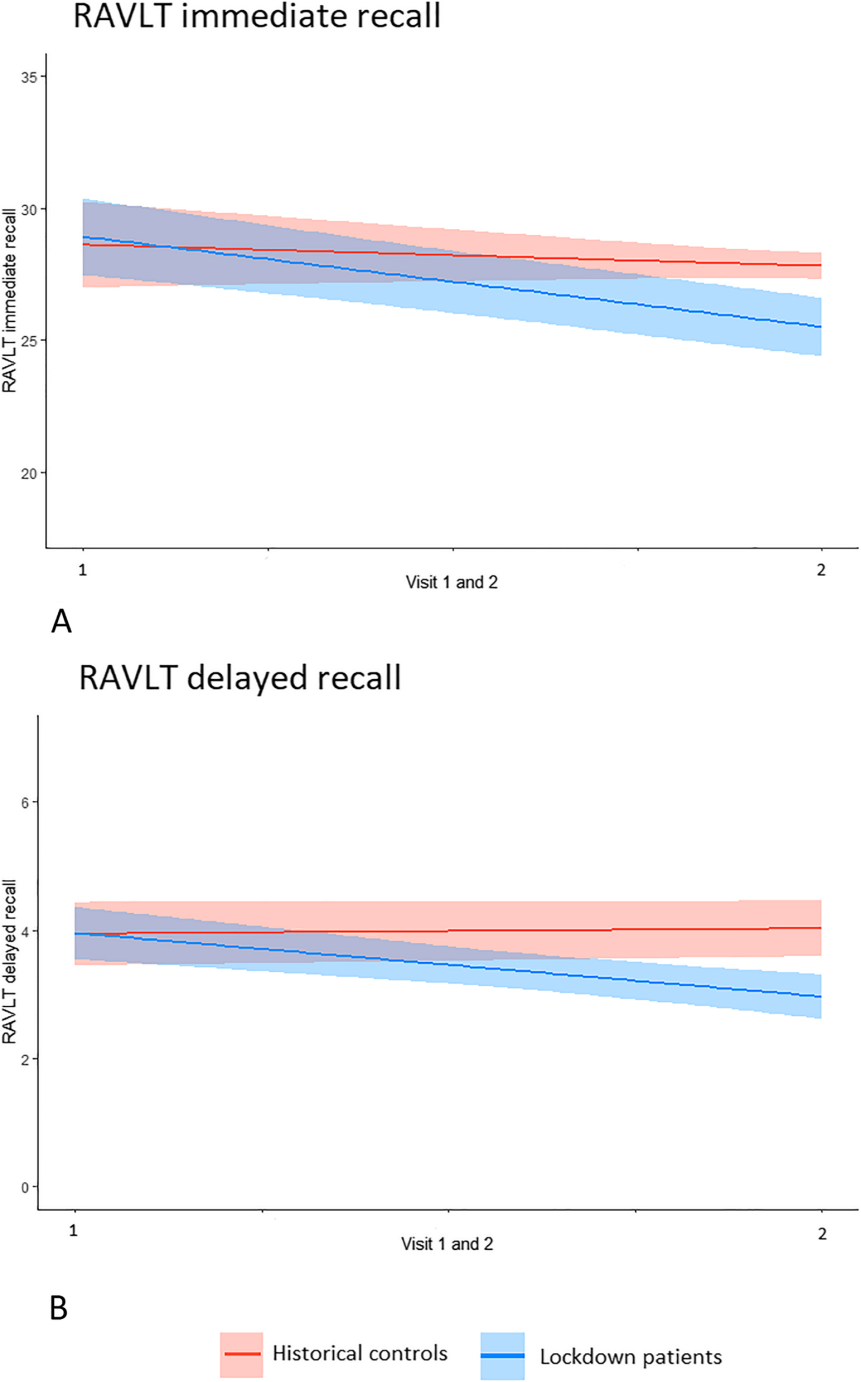
RAVLT immediate (**A**) and delayed (**B**) recall trajectories in lockdown patients compared to historical controls. RAVLT, Rey-Auditory Verbal Learning Test

Table 2 shows changes in cognitive test scores over time between lockdown patients and historical controls. As expected and illustrating successful matching, there was no effect of group on baseline test performance for any of the tests. By contrast, we found significant interactions between time and group for RAVLT immediate recall [*B*(SE) =  − 2.62 (1.07), *p* = 0.015] and delayed recall [*B*(SE) =  − 1.07 (0.34), *p* = 0.002], indicating faster memory decline during lockdown; see Fig. 2. There were no significant interactions between time and group for MMSE, TMT part A and B, and category fluency. Excluding patients who reported a COVID-19 infection and their matched historical controls did not change the results. Mean cognitive test scores of visit 1 and visit 2 can be found in the supplemental material (Table e-4 in the supplement). We found similar results, i.e., faster decline on immediate and delayed recall, and faster decline in category fluency in lockdown patients when LMM analyses were repeated in the unmatched cohort (see Table e-5 in the supplement).

**Table 2:** Change in cognitive test scores over time between lockdown patients and matched historical controls

		**MMSE**	**TMT part A**	**TMT part B**	**RAVLT immediate recall**	**RAVLT delayed recall**	**Category fluency**
		*B* (SE)	*B* (SE)	*B* (SE)	*B* (SE)	*B* (SE)	*B* (SE)
All diagnoses	Group	− 0.26 (0.57)	− 5.23 (8.36)	− 12.02 (13.66)	0.31 (1.59)	0.01 (0.49)	0.83 (0.86)
	Time × Group	0.52 (0.46)	− 1.57 (6.18)	− 1.92 (8.84)	− 2.62 (1.07)*	− 1.07 (0.34)**	− 0.61 (0.62)
SCD	Group	− 0.22 (0.59)	5.88 (4.53)	39.03 (18.94)*	− 1.65 (4.25)	0.28 (1.32)	0.33 (1.90)
	Time × Group	− 0.72 (0.64)	− 2.77 (2.88)	− 4.11 (9.25)	− 6.85 (2.97)*	− 2.45 (1.11)*	0.23 (1.91)
MCI	Group	− 0.39 (0.72)	− 5.59 (6.36)	− 5.91 (18.13)	3.17 (2.28)	0.69 (0.67)	0.61 (1.22)
	Time × Group	− 0.10 (0.62)	3.81 (5.38)	− 3.95 (13.42)	− 6.14 (1.78)**	− 1.50 (0.51)**	0.03 (1.03)
Dementia	Group	− 0.11 (0.85)	− 12.22 (15.02)	− 40.39 (24.18)	0.01 (1.74)	− 0.16 (0.56)	1.07 (1.17)
	Time × Group	1.24 (0.79)	− 2.15 (11.96)	− 6.25 (20.24)	0.96 (1.56)	− 0.47 (0.53)	− 0.39 (0.80)

Model: Group (0 = historical controls, 1 = lockdown patients), and interaction Time × Group

*MMSE* Mini-Mental State Examination, *MCI* Mild cognitive impairment, *TMT* Trail Making Test, *RAVLT* Rey-Auditory Verbal Learning Test, *SCD* Subjective cognitive decline

^*^p < .05

^**^p < .01

Subsequently, we stratified by syndrome diagnosis and found that the effect of lockdown measures on memory was mostly attributable to non-demented patients. Interactions between time and group for SCD and MCI were significant, but not for dementia on RAVLT immediate recall [SCD: *B*(SE) =  − 6.85 (2.97), *p* = 0.027; MCI: *B*(SE) =  − 6.14 (1.78), *p* = 0.001] and delayed recall [SCD: *B*(SE) =  − 2.45 (1.11), *p* = 0.035; MCI: *B*(SE) =  − 1.50 (0.51), *p* = 0.005]; see Table 2 (demographic characteristics stratified by syndrome diagnosis are summarized in Table e-6). There were no significant interactions between time and group on any other cognitive outcome measure.

Finally, we performed a sensitivity analysis, restricted to amyloid-positive patients only. We found similar results, i.e., faster decline on immediate and delayed recall, in lockdown patients [RAVLT immediate recall: *B*(SE) =  − 3.91 (1.84), *p* = 0.037; RAVLT delayed recall: *B*(SE) =  − 2.18 (0.63), *p* < 0.001].

## Discussion

We showed that cognitive decline, specifically in memory, was faster during COVID-19 lockdown than usually. Especially pre-dementia (SCD and MCI) patients were more prone to faster memory decline during lockdown. This provides evidence for the notion that COVID-19 lockdown regulations further increased the risk of progression in patients at risk of dementia.

Our results add important evidence to current literature on cognitive decline in times of COVID-19 lockdown. Former studies have suggested that patients, but also cognitively healthy individuals, experienced more cognitive failures during lockdown. However, this was self-reported in survey studies and not measured by objective cognitive testing [3, 4, 9]. In these survey studies, the most frequently reported cognitive failures were associated with memory function [9]. Two small studies objectively tested cognition and showed worse cognitive outcome in memory clinic patients during lockdown [10, 11], and specifically in memory function [10]. However, these two studies did not have a (matched) control group for comparison. Our results, using carefully matched groups and by means of standardized neuropsychological tests, show that the effect was most prominent on memory and in patients with SCD and MCI, rather than dementia.

Episodic memory is the most prominently impaired cognitive domain in early AD [26, 27]. In addition, the association between amyloid and hippocampal atrophy are early biomarkers of AD [27]. As such, it is plausible that the observed effect in the present study was in the episodic memory domain. This also fits with the findings of the sensitivity analysis where we restricted the analysis to amyloid positives only and found similar or even somewhat more pronounced results. An alternative explanation is that tests for memory, such as the RAVLT, have better test properties in terms of normal distribution, validity, and responsiveness to change than tests for other cognitive domains, and this could also contribute to our finding.

In a large proportion of MCI patients, the underlying cause of cognitive complaints is Alzheimer’s disease [5]. The loss of structure in combination with a stressful situation such as COVID-19 pandemic could have caused the disease to clinically unfold sooner than it would have done otherwise. This line of reasoning may even hold for some of the individuals with SCD, where the lockdown measures could have contributed to clinical manifestation of the underlying disease [6]. When we stratified our analysis for syndrome, we indeed found that findings were strongest for individuals with MCI and SCD. Furthermore, poor social activity is a risk factor for cognitive decline and dementia [28–30]. It is conceivable that lockdown restrictions had impact on social activity, particularly in patients with SCD and MCI. Contrary to former studies, we did not find an effect of lockdown measures on cognitive decline in dementia patients. There are a number of potential explanations; firstly, patients with dementia may have already functioned at a bottom level at baseline, obscuring a (faster) decline in cognitive test scores over time. Secondly, in dementia patients, faster progression could manifest in non-cognitive signs and symptoms, instead of cognitive decline. In previous survey studies, more behavioral symptoms in memory clinic patients (i.e., apathy, changes in sleeping behavior, repetitive behavior, and aggression) were reported during lockdown by a great amount of caregivers, and even a small proportion of patients themselves [3, 4]. Finally, it is conceivable that the dementia patients experiencing fastest progression did not come back for repeated assessment to the clinic, due to institutionalization or mortality. COVID-19 lockdown might also have affected the mortality rate and causes of death in this vulnerable group of patients. Future research is necessary to study whether COVID-19 lockdown affected mortality rate in this group of patients, for example by linking to external registries in the Netherlands (e.g., Statistics Netherlands).

In previous survey studies, caregivers reported worries for faster cognitive decline in patients with dementia [3, 4]. It is possible that these worries reflect increased caregiver burden during COVID-19 lockdown, rather than actual decline. Along another line of reasoning, it could be the case that patients with dementia were less aware of the disturbance in daily life caused by the COVID-19 restrictions, because of less general consciousness due to their illness, or experienced less stress during lockdown than pre-dementia patients [31, 32].

Among the limitations of the current study is that all patients were included in a tertiary memory clinic, which might not give a general representation of memory clinic patients in the Netherlands. However, our mixed sample included SCD, MCI, and dementia patients and thus represents the full cognitive continuum. Another potential limitation is potential selection bias, as lockdown patients were pre-screened by their assessing physician via telephone before their second visit, due to COVID-19 measures. The assessing physician decided whether it was necessary to plan a physical visit, which may have resulted in a different mix of patients visiting our center for follow-up than before COVID-19. Nonetheless, the indication for a visit varied greatly; from patient’s and caregiver’s questions about care and support network, a physical visit at the request of patient or caregiver, to suspicion of cognitive decline by the physician. Additionally, reasons for not returning to the clinic after 1 year may have been different for the lockdown and the historical control groups. For example, more people may have died or been seriously ill during follow-up in the lockdown group (due to COVID-19 infections). This may have led to an underestimation of the effect. This pre-screening and attrition might have caused a potential selection bias, which we have attempted to mitigate by our careful matching procedure, including the variables diagnosis and MMSE score. Furthermore, information on COVID-19 infection among patients during the pandemic was only available by self-report. There might be an underrepresentation of the actual COVID-19 infections among our patients. However, it will be unlikely that many patients experienced a COVID-19 infection without noticing this, especially in the first year of the pandemic when the COVID-19 virus caused more severe symptoms than later in the pandemic. Lastly, important to acknowledge is the variability in COVID-19 lockdowns between different countries. As different restrictions against COVID-19 were issued across countries worldwide, comparison with studies on cognitive decline during lockdown in other countries must be done with caution. Nonetheless, the impact of COVID-19 pandemic on patients’ lives may be similar to a large extent.

Among the strengths of this study is our careful statistical approach, using optimal Mahalanobis distance matching, in which we matched patients during COVID-19 lockdown to historical controls. The outcome of the matching analysis had led to well-balanced groups between lockdown patients and historical controls. In addition, we used standardized cognitive tests to assess longitudinal cognitive decline. The balanced groups and longitudinal cognitive data allowed us to conclude that the general annual decline during COVID-19 was more severe than before COVID-19 times, and this cannot be attributed to COVID-19 itself, but rather to the large impact of the lockdown measures on society. In addition, our sample sizes of lockdown patients and historical controls were of adequate size, with over one hundred patients in both groups. Furthermore, we were able to stratify by syndrome diagnosis, while maintaining balance between lockdown patients and historical controls.

## Conclusion

In conclusion, we provided evidence suggesting that memory clinic patients show faster decline in memory function during COVID-19 lockdown than before, indicating that COVID-19 lockdown regulations contributed to faster cognitive decline. The results of the present study suggest that it is important that social contact, (in)formal support, and care continue, and social networks remain available for memory clinic patients during times of lockdown to prevent faster cognitive decline. This means that social contact, support, and care for these patients should only be disrupted when good alternatives can be offered, and good quality support can still be guaranteed. It is recommendable to develop protocols for remaining social contact, and good quality alternative care and support in times of restrictive measures, when there is a risk of social isolation, and disruption of services and support. Moreover, focus of continuing good quality support should specifically be on pre-dementia patients, as this patient group is more prone for faster cognitive decline.

## Supplementary Information

**Additional file 1: Table e-1. **Demographic characteristics of historical control patients. **Table e-2.** Balance before matching for age, MMSE and time between visit 1 and 2. **Table e-3.** Balance before matching for sex, diagnosis and type visit 1. **Figure e-1.** Balance before matching of age in years at visit 1 in lockdown patients and historical controls. **Figure e-2.** Balance before matching of MMSE (Mini-Mental State Examination) at visit 1 in lockdown patients and historical controls. **Figure e-3.** Balance before matching of time between visit 1 and 2 in months in lockdown patients and historical controls. **Table e-4.** Mean cognitive test scores of lockdown patients and matched historical controls at visit 1 and visit 2. **Table e-5.** Change in cognitive test scores over time between lockdown patients and all historical controls (n = 640). **Table e-6.** Demographic characteristics of lockdown patients and matched historical controls, stratified by syndrome diagnosis: SCD, MCI and dementia.

## Data Availability

The dataset used and/or the analysis performed can be provided upon reasonable request.

## References

[1] Douglas M, Katikireddi SV, Taulbut M, McKee M, McCartney G (2020). Mitigating the wider health effects of covid-19 pandemic response. BMJ.

[2] Iodice F, Cassano V, Rossini PM (2021). Direct and indirect neurological, cognitive, and behavioral effects of COVID-19 on the healthy elderly, mild-cognitive-impairment, and Alzheimer’s disease populations. Neurol Sci.

[3] van Maurik IS, Bakker ED, van den Buuse S, Gillissen F, van de Beek M, Lemstra E (2020). Psychosocial effects of corona measures on patients with dementia, mild cognitive impairment and subjective cognitive decline. Front Psychiatry.

[4] Bakker ED, van Maurik IS, Mank A, Zwan MD, Waterink L, van den Buuse S (2022). Psychosocial effects of COVID-19 measures on (pre-)dementia patients during second lockdown. J Alzheimers Dis.

[5] Petersen RC, Smith GE, Waring SC, Ivnik RJ, Tangalos EG, Kokmen E (1999). Mild cognitive impairment: clinical characterization and outcome. Arch Neurol.

[6] Slot RER, Sikkes SAM, Berkhof J, Brodaty H, Buckley R, Cavedo E (2019). Subjective cognitive decline and rates of incident Alzheimer’s disease and non-Alzheimer’s disease dementia. Alzheimers Dement.

[7] Manly JJ, Tang MX, Schupf N, Stern Y, Vonsattel JP, Mayeux R (2008). Frequency and course of mild cognitive impairment in a multiethnic community. Ann Neurol.

[8] Ravaglia G, Forti P, Montesi F, Lucicesare A, Pisacane N, Rietti E (2008). Mild cognitive impairment: epidemiology and dementia risk in an elderly Italian population. J Am Geriatr Soc.

[9] Maggi G, Baldassarre I, Barbaro A, Cavallo ND, Cropano M, Nappo R (2021). Mental health status of Italian elderly subjects during and after quarantine for the COVID-19 pandemic: a cross-sectional and longitudinal study. Psychogeriatrics.

[10] Ismail II, Kamel WA, Al-Hashel JY (2021). Association of COVID-19 pandemic and rate of cognitive decline in patients with dementia and mild cognitive impairment: a cross-sectional study. Gerontol Geriatr Med.

[11] Tondo G, Sarasso B, Serra P, Tesser F, Comi C (2021). The impact of the COVID-19 pandemic on the cognition of people with dementia. Int J Environ Res Public Health.

[12] van der Flier WM, Pijnenburg YA, Prins N, Lemstra AW, Bouwman FH, Teunissen CE (2014). Optimizing patient care and research: the Amsterdam Dementia Cohort. J Alzheimers Dis.

[13] van der Flier WM, Scheltens P (2018). Amsterdam Dementia Cohort: performing research to optimize care. J Alzheimers Dis.

[14] Albert MS, DeKosky ST, Dickson D, Dubois B, Feldman HH, Fox NC (2011). The diagnosis of mild cognitive impairment due to Alzheimer's disease: recommendations from the National Institute on Aging-Alzheimer’s Association workgroups on diagnostic guidelines for Alzheimer’s disease. Alzheimers Dement.

[15] McKeith IG, Dickson DW, Lowe J, Emre M, O'Brien JT, Feldman H (2005). Diagnosis and management of dementia with Lewy bodies: third report of the DLB Consortium. Neurology.

[16] McKeith IG, Boeve BF, Dickson DW, Halliday G, Taylor JP, Weintraub D (2017). Diagnosis and management of dementia with Lewy bodies: Fourth consensus report of the DLB Consortium. Neurology.

[17] Rascovsky K, Hodges JR, Knopman D, Mendez MF, Kramer JH, Neuhaus J (2011). Sensitivity of revised diagnostic criteria for the behavioural variant of frontotemporal dementia. Brain.

[18] Gorno-Tempini ML, Hillis AE, Weintraub S, Kertesz A, Mendez M, Cappa SF (2011). Classification of primary progressive aphasia and its variants. Neurology.

[19] Jessen F (2014). Subjective and objective cognitive decline at the pre-dementia stage of Alzheimer’s disease. Eur Arch Psychiatry Clin Neurosci.

[20] Reitan RM (1958). Validity of the trail making test as an indicator of organic brain damage. Percept Mot Skills.

[21] Saan RJ, Deelman BG (1986). De 15-Woordentests A en B. Een voorlopige handleiding (Intern rapport).

[22] Van der Elst W, Van Boxtel MP, Van Breukelen GJ, Jolles J (2006). Normative data for the Animal, Profession and Letter M Naming verbal fluency tests for Dutch speaking participants and the effects of age, education, and sex. J Int Neuropsychol Soc.

[23] Ho D, Imai K, King G, Stuart EA (2011). MatchIt: nonparametric preprocessing for parametric causal inference. J Stat Softw.

[24] Stuart EA (2010). Matching methods for causal inference: a review and a look forward. Stat Sci.

[25] Gu XS, Rosenbaum PR (1993). Comparison of multivariate matching methods: structures, distances, and algorithms. J Comput Graph Stat.

[26] Dubois B, Feldman HH, Jacova C, Hampel H, Molinuevo JL, Blennow K (2014). Advancing research diagnostic criteria for Alzheimer’s disease: the IWG-2 criteria. Lancet Neurol.

[27] Tomadesso C, de La Sayette V, de Flores R, Bourgeat P, Villemagne VL, Egret S (2018). Neuropsychology and neuroimaging profiles of amyloid-positive versus amyloid-negative amnestic mild cognitive impairment patients. Alzheimers Dement (Amst).

[28] Kuiper JS, Zuidersma M, Oude Voshaar RC, Zuidema SU, van den Heuvel ER, Stolk RP (2015). Social relationships and risk of dementia: a systematic review and meta-analysis of longitudinal cohort studies. Ageing Res Rev.

[29] James BD, Wilson RS, Barnes LL, Bennett DA (2011). Late-life social activity and cognitive decline in old age. J Int Neuropsychol Soc.

[30] Stewart CC, Yu L, Glover CM, Mottola G, Bennett DA, Wilson RS (2020). Loneliness interacts with cognition in relation to healthcare and financial decision making among community-dwelling older adults. Gerontologist.

[31] Jenkins A, Tree J, Tales A (2021). Distinct profile differences in subjective cognitive decline in the general public are associated with metacognition, negative affective symptoms, neuroticism, stress, and poor quality of life. J Alzheimers Dis.

[32] Elfgren C, Gustafson L, Vestberg S, Passant U (2010). Subjective memory complaints, neuropsychological performance and psychiatric variables in memory clinic attendees: a 3-year follow-up study. Arch Gerontol Geriatr.

