# Supplementary material for: Steeper memory decline after COVID-19 lockdown measures
Source: Alzheimers Res Ther. 2023 Apr 15;15:81. doi: 10.1186/s13195-023-01226-5 (PMC10104769; doi:10.1186/s13195-023-01226-5)
Supplement: Supplementary file 1 — Additional file 1: Table e-1. Demographic characteristics of historical control patients. Table e-2. Balance before matching for age, MMSE and time between visit 1 and 2. Table e-3. Balance before matching for sex, diagnosis and type visit 1. Figure e-1. Balance before matching of age in years at visit 1 in lockdown patients and historical controls. Figure e-2. Balance before matching of MMSE (Mini-Mental State Examination) at visit 1 in lockdown patients and historical controls. Figure e-3. Balance before matching of time between visit 1 and 2 in months in lockdown patients and historical controls. Table e-4. Mean cognitive test scores of lockdown patients and matched historical controls at visit 1 and visit 2. Table e-5. Change in cognitive test scores over time between lockdown patients and all historical controls (n = 640). Table e-6. Demographic characteristics of lockdown patients and matched historical controls, stratified by syndrome diagnosis: SCD, MCI and dementia. [file 13195_2023_1226_MOESM1_ESM.docx]

**Supplemental material**

**Table e-1.** Demographic characteristics of historical control patients

|  |  | **Historical control patients** |
| --- | --- | --- |
|  |  | n=640 (100%) |
| Age in years | n=640 | 65 ± 8 |
| Sex, female | n=640 | n=265 (41%) |
| MMSE at visit 1 | n=640 | 26 ± 4 |
| Years of education | n=544 | 12 ± 3 |
| Diagnosis at visit 1 | n=640 |  |
| SCD |  | n=236 (37%) |
| MCI |  | n=139 (22%) |
| AD |  | n=180 (28%) |
| FTD or PPA |  | n=24 (4%) |
| DLB |  | n=41 (6%) |
| Postponed diagnosis |  | n=20 (3%) |
| Visit 1 type | n=640 |  |
| Baseline |  | n=301 (47%) |
| Follow-up |  | n=339 (53%) |
| Time between visit 1 and visit 2  (in years) | n=640 | 1.1 ± 0.2 |
|  |  |  |

AD: Alzheimer’s dementia, DLB: dementia with Lewy bodies, FTD: frontotemporal dementia, MCI: mild cognitive impairment, MMSE: Mini-Mental State Examination, PPA: primary progressive aphasia, SCD: subjective cognitive decline.

| **Table e-2.** Balance before matching for age, MMSE and time between visit 1 and 2. | | | | | |  |  |
| --- | --- | --- | --- | --- | --- | --- | --- |
|  |  | Min. | 1st quartile | Median | Mean | 3rd quartile | Max. |
| Age in years | Historical controls | 41 | 59 | 66 | 65 | 71 | 83 |
|  | Lockdown patients | 47 | 62 | 66 | 66 | 72 | 89 |
| MMSE at visit 1 | Historical controls | 12 | 24 | 27 | 26 | 29 | 30 |
|  | Lockdown patients | 12 | 23 | 26 | 25 | 28 | 30 |
| Time between visit 1 and 2 | Historical controls | 4 | 11 | 12 | 13 | 14 | 23 |
|  | Lockdown patients | 5 | 12 | 13 | 14 | 16 | 24 |

MMSE: Mini-Mental State Examination.

| **Table e-3.** Balance before matching for sex, diagnosis and type visit 1. | | |
| --- | --- | --- |
|  | Lockdown patients | Historical controls |
|  | n = 113 (100%) | n = 640 (100%) |
| Sex, males | n = 79 (70%) | n = 375 (59%) |
| Sex, females | n = 34 (30%) | n = 265 (41%) |
| Diagnosis, SCD | n = 18 (16%) | n = 236 (37%) |
| Diagnosis, MCI | n = 31 (27%) | n = 139 (22%) |
| Diagnosis, AD | n = 26 (23%) | n = 180 (28%) |
| Diagnosis, DLB | n = 23 (20%) | n = 41 (6%) |
| Diagnosis, FTD/PPA | n = 6 (5%) | n = 24 (4%) |
| Diagnosis, postponed | n = 9 (8%) | n = 20 (3%) |
| Type visit 1, baseline | n= 36 (32%) | n = 301 (47%) |
| Type visit 1, follow-up | n = 77 (68%) | n = 339 (53%) |

SCD: subjective cognitive decline, MCI: mild cognitive impairment, AD: Alzheimer’s dementia, FTD: frontotemporal dementia, PPA: primary progressive aphasia, DLB: dementia with Lewy bodies.


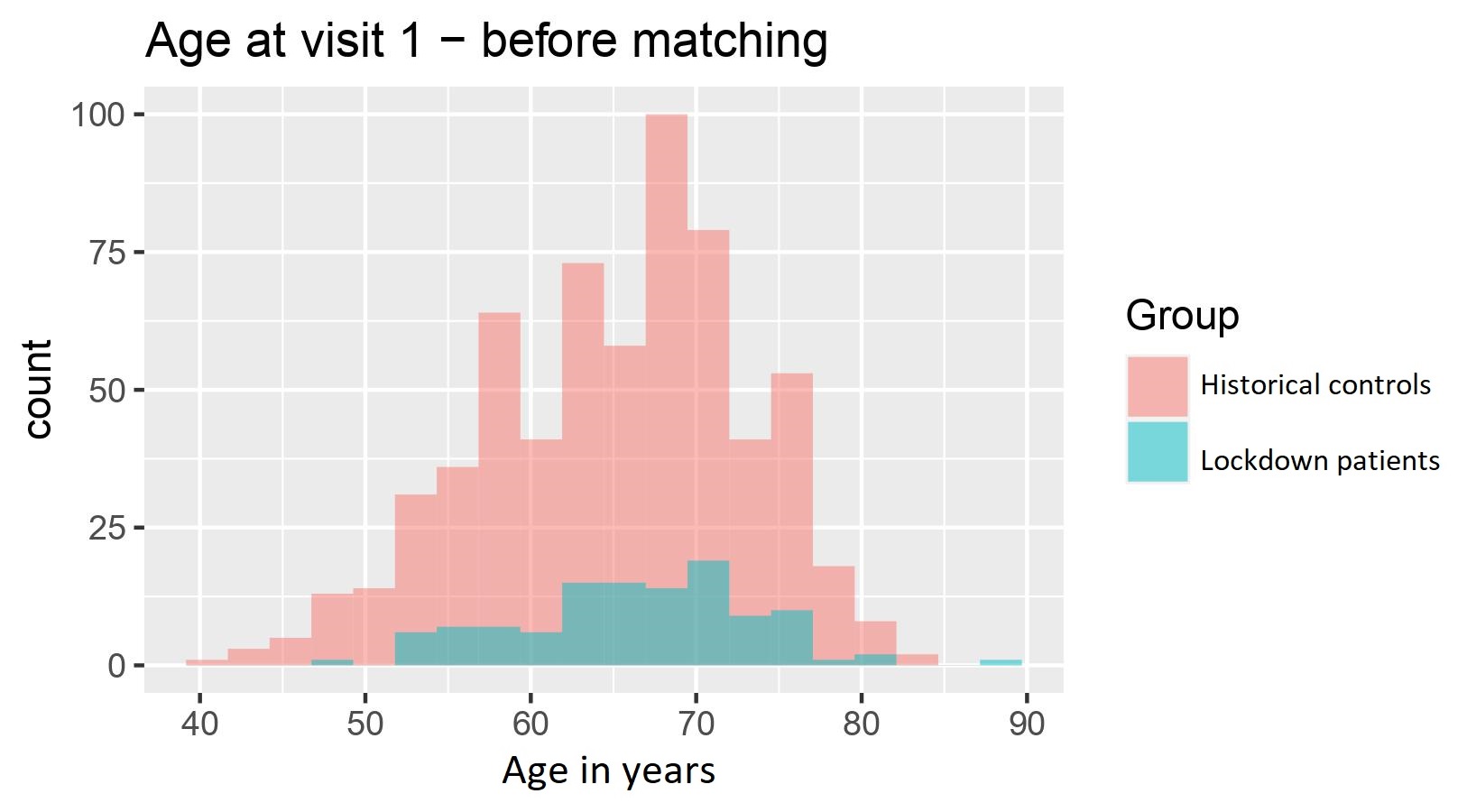


**Figure e-1.** Balance before matching of age in years at visit 1 in lockdown patients and historical controls


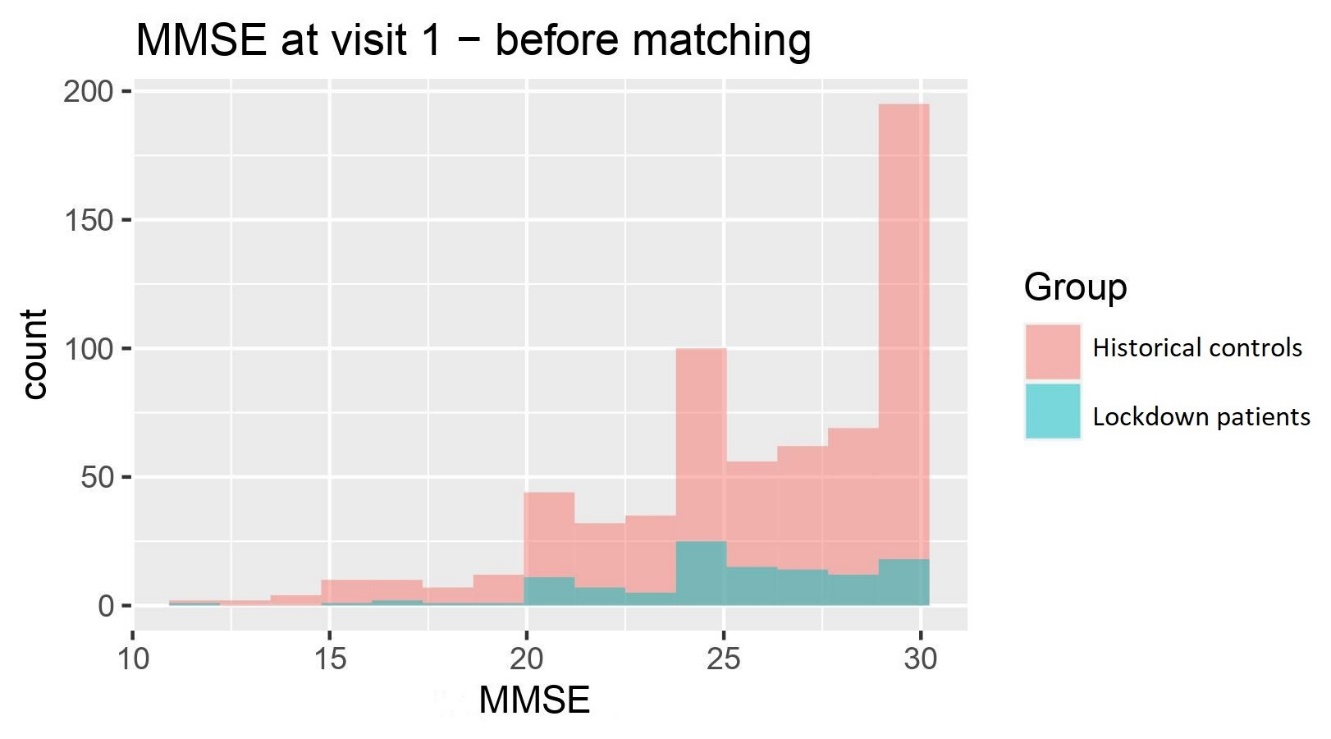


**Figure e-2.** Balance before matching of MMSE (Mini-Mental State Examination) at visit 1 in lockdown patients and historical controls

**
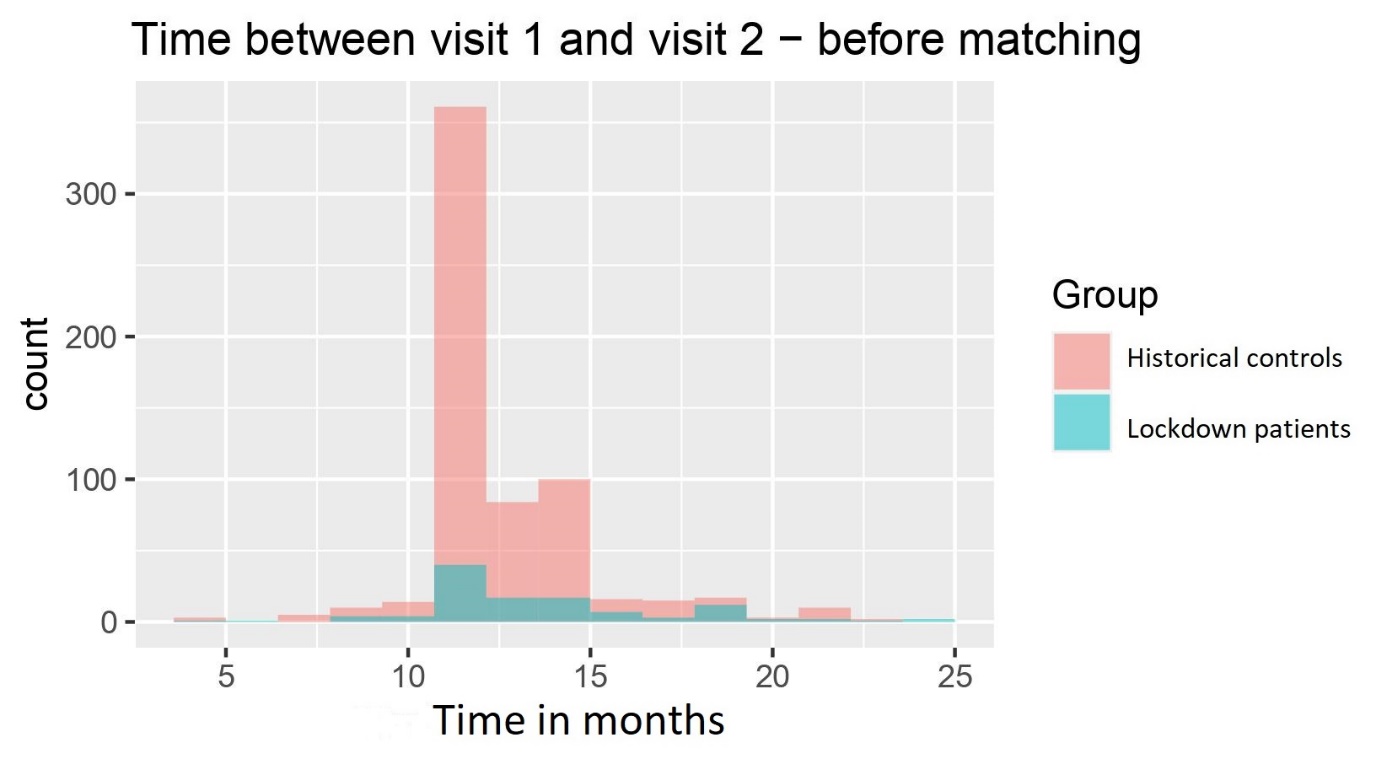
**

**Figure e-3.** Balance before matching of time between visit 1 and 2 in months in lockdown patients and historical controls

| **Table e-4.** Mean cognitive test scores of lockdown patients and matched historical controls at visit 1 and visit 2 | | | | | |
| --- | --- | --- | --- | --- | --- |
|  |  | **Lockdown patients** | | **Matched historical controls** | |
|  |  | Visit 1 | Visit 2 | Visit 1 | Visit 2 |
| MMSE | M ± SD | 25 ± 3 | 24 ± 4 | 25 ± 3 | 24 ± 6 |
| TMT part A | M ± SD | 59 ± 48 | 71 ±60 | 63 ± 53 | 79 ± 77 |
| TMT part B | M ± SD | 134 ± 76 | 138 ± 81 | 147 ± 88 | 148 ± 98 |
| RAVLT immediate recall | M ± SD | 29 ± 11 | 26 ± 10 | 29 ± 12 | 28 ± 14 |
| RAVLT delayed recall | M ± SD | 4 ± 4 | 3 ± 3 | 4 ± 4 | 4 ± 4 |
| Category fluency | M ± SD | 18 ± 6 | 16 ± 6 | 17 ± 7 | 16 ± 6 |
| MMSE: Mini-Mental State Examination, TMT: Trail Making Test, RAVLT: Rey-Auditory Verbal Learning Test. | | | | | |

| **Table e-5.**  Change in cognitive test scores over time between lockdown patients and all historical controls (n = 640) | | | | | | |
| --- | --- | --- | --- | --- | --- | --- |
|  | **MMSE** | **TMT part A** | **TMT part B** | **RAVLT immediate recall** | **RAVLT delayed recall** | **Category fluency** |
|  | *B* (SE) | *B* (SE) | *B* (SE) | *B* (SE) | *B* (SE) | *B* (SE) |
| Group | -0.55 (0.46) | 0.67 (6.31) | 7.06 (10.65) | -2.69 (1.39) | -0.88 (0.43)* | -0.57 (0.75) |
| Time * Group | -0.24 (0.31) | 4.94 (4.75) | 1.47 (6.86) | -3.26 (0.80)*** | -0.93 (0.23)*** | -1.37 (0.51)** |
| *p<.05, **p<.01, ***p<.001 | |  |  |  |  |  |
| Model: Time (0 = visit 1, 1 = visit 2), Group (0 = historical controls, 1 = lockdown patients), and interaction Time * Group. MMSE: Mini-Mental State Examination, TMT: Trail Making Test, RAVLT: Rey-Auditory Verbal Learning Test. | | | | | | |

| **Table e-6.** Demographic characteristics of lockdown patients and matched historical controls, stratified by syndrome diagnosis: SCD, MCI and dementia | | | | | | |
| --- | --- | --- | --- | --- | --- | --- |
|  | **SCD** |  | **MCI** |  | **Dementia** |  |
|  | Lockdown patients (n = 18) | Matched historical controls (n = 18) | Lockdown patients (n = 31) | Matched historical controls (n = 31) | Lockdown patients (n = 55) | Matched historical controls (n = 55) |
| Age in years | 65 ± 8 | 64 ± 8 | 67 ±7 | 67 ± 7 | 67 ± 8 | 67 ± 7 |
| Sex, female | n=2 (11%) | n=2 (11%) | n=14 (45%) | n=14 (45%) | n=16 (29%) | n=16 (29%) |
| MMSE visit 1 | 29 ± 1 | 29 ± 1 | 26 ± 2 | 26 ± 2 | 24 ± 4 | 24 ± 4 |
| Years of education | 13 ± 3 | 13 ± 3 | 12 ± 3 | 12 ± 3 | 12 ± 3 | 11 ± 2 |
| Visit type 1 |  |  |  |  |  |  |
| Baseline | n=4 (22%) | n=4 (22%) | n=9 (29%) | n=9 (29%) | n=17 (31%) | n=18 (33%) |
| Follow-up | n=14 (78%) | n=14 (78%) | n=22 (71%) | n=22 (71%) | n=38 (69%) | n=37 (67%) |
| Time between V1 and V2  (in months) | 14 ± 3 | 13 ± 3 | 13 ± 2 | 13 ± 3 | 14 ± 4 | 13 ± 3 |

MCI: mild cognitive impairment, MMSE: Mini-Mental State Examination, SCD: subjective cognitive decline.
